# Capillary Electrophoresis as a Useful Tool to Separate Isomeric Opioid–Neurotensin Hybrid Peptides

**DOI:** 10.3390/molecules30214186

**Published:** 2025-10-26

**Authors:** Joanna Zdunek, Patrycja Kleczkowska, Łukasz Szeleszczuk, Wojciech Kamysz, Karol Sikora, Błażej Grodner

**Affiliations:** 1 Department of Biochemistry and Pharmacogenomics, Medical University of Warsaw, 1 Banacha Str., 02-097 Warsaw, Poland; s086155@student.wum.edu.pl; 2 Maria Sklodowska-Curie Medical Academy in Warsaw, 03-411 Warsaw, Poland; hazufiel@wp.pl; 3 Department of Biomedical Research, National Medicines Institute, 00-725 Warsaw, Poland; 4 Department of Organic and Physical Chemistry, Medical University of Warsaw, 1 Banacha Str., 02-097 Warsaw, Poland; lukasz.szeleszczuk@wum.edu.pl; 5 Department of Inorganic Chemistry, Faculty of Pharmacy, Medical University of Gdansk, 80-416 Gdansk, Poland; wojciech.kamysz@gumed.edu.pl (W.K.); karol.sikora@gumed.edu.pl (K.S.)

**Keywords:** hybrid peptide, neurotensin, opioid, capillary electrophoresis

## Abstract

We developed and validated a capillary electrophoresis (CE) method for the separation of two opioid–neurotensin hybrid peptides, recently presented as potent analgesics being decapeptides with a hybridic nature (i.e., H-Dmt-D-Lys-Phe-Phe-Lys-Lys-Pro-Phe-Tle-Leu-OH; PK20 and its structural analogue H-Dmt-D-Lys-Phe-Phe-Lys-Lys-Pro-Phe-Ile-Leu-OH; [Ile9]PK20). As these two chimeras differ by only one amino acid, Tle→Ile, and are characterized by possessing the same molecular weight while having different spatial conformations, the aim of the study was to determine their potential separation in terms of the presence of any differences resulting from this structural modification. The separation process was performed using an eCAP fused silica capillary at a detection wavelength of 200 nm in 25 mM phosphate buffer at pH 2.5. The analysis was performed at 25 °C and 10 kV. The developed method was validated by assessing linearity in the concentration range from 50 to 5000 ng/mL. Very good linearity was obtained, with the coefficient of determination (R^2^) ranging from 0.9991 to 0.9999 for both analyzed derivatives. The method demonstrated baseline resolution (Rs = 1.4). The limit of quantification ranged from 34.72 ng/mL to 34.98 ng/mL. The recoveries of all derivatives ranged from 94.8% to 100%. The total analysis time was only 6 min. The developed method enables the determination of PK20 and [Ile9]PK20 derivatives both in aqueous solutions and in serum.

## 1. Introduction

Peptides exhibit many useful properties for human health, including antimicrobial, antifungal, antiviral, and anticancer activities. Some of them have been isolated from natural sources; a good example of this is insulin [1]. However, currently, substantial progress has been made in the field of peptide synthesis in addition to natural peptides [2]. Notably, these novel peptides and peptide-based compounds are known to be composed of amino acids whose structures go beyond those of the known 20 naturally existing amino acids. Therefore, they not only mimic natural structures but are characterized with a better pharmacokinetic and pharmacodynamic profile, including an improved safety profile. In this context, it can be freely said that peptide drug development has recently made great progress and now is one of the main topics in pharmacy and medicine.

Among peptide structures, significant interest is given to hybrid compounds (termed also as chimeras, bivalent ligands, or multiple ligands) [3], which are suggested to cover multiple therapeutic areas. Indeed, by combining at least two different molecules with a diverse mechanism of action, a multitarget and thus multifunctional molecule can be obtained [4,5,6,7]. These specific structures can offer an advantage over the traditional and still popular polypharmaceutical approach, as they demonstrate a significant reduction in the number and intensity of clinically important side effects, including ADME-related pharmacokinetic limitations [8]. Moreover, a mechanism that allows for simultaneous interaction with molecular targets is switched on for such compounds.

**Figure 1:**
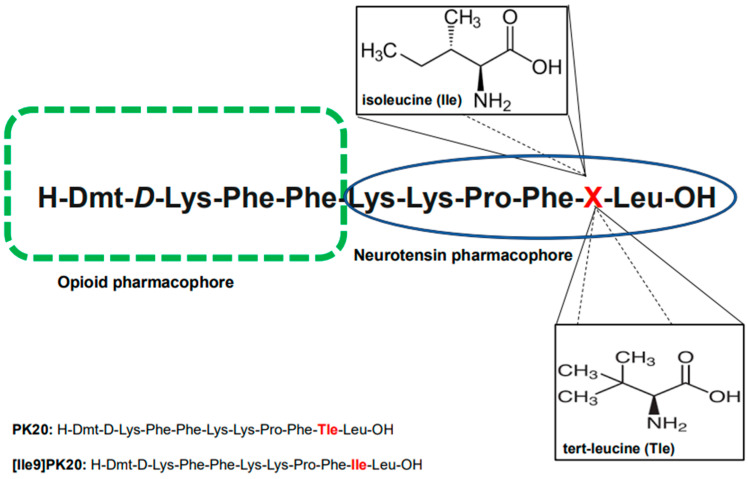
Chemical structures of the tested opioid–neurotensin hybrid peptides, PK20 and [Ile9]PK20, with the indication of pharmacophores. The differences in the amino acid structure of both compounds are marked in red.

Nevertheless, considering the importance and usefulness of peptide drugs, methodologies for their deep investigation are strongly needed. In this context, we aimed to present the effectiveness of a novel capillary electrophoresis technique (CE) used to determine both the qualitative and quantitative presence of two recently synthesized hybrid peptides in biological material (serum) and aqueous solutions. Importantly, these two chimeras (H-Dmt-D-Lys-Phe-Phe-Lys-Lys-Pro-Phe-**Tle**-Leu-OH; PK20 and H-Dmt-D-Lys-Phe-Phe-Lys-Lys-Pro-Phe-**Ile**-Leu-OH; [Ile9]PK20) are known to possess only one modification with tert-leucine (L-Tle) replaced with its isomer, isoleucine (L-Ile); the molecular formula of both is C_6_H_13_NO_2_ (*m*/*z* 131) (Figure 1). As this modification was found to significantly affect the agonist/antagonist behavior of the compound towards its receptor targets (i.e., mu opioid receptor MOR and neurotensin NTS) as well as the pharmacokinetic profile [5,8], it does not change the molecular weight of the molecules tested. The opioid–neurotensin hybrids PK20 and [Ile9]PK20 are promising analgesics with distinct receptor profiles [5,8]. They differ only at position 9 (Tle vs. Ile), yet this modification influences receptor affinity and pharmacodynamics. Since both molecules have identical molecular weights, separation is challenging using conventional LC or LC-MS/MS methods, which rely on mass differences [9]. Capillary electrophoresis (CE), based on the charge-to-size ratio, offers high efficiency for distinguishing isomers with subtle conformational differences [10]. The aim of this study was to develop and validate a CE method for the separation and quantification of PK20 and [Ile9]PK20 in aqueous solutions and serum. Unlike previous chromatographic approaches, we demonstrate CE’s ability to resolve these isomeric peptides rapidly and reproducibly. While this study focuses on method development, future work will integrate mass spectrometric confirmation and validation across multiple biological matrices.

## 2. Results and Discussion

### 2.1. Optimization

In order to develop a method for the quantitative determination of [Ile9]PK20 and PK20 compounds using capillary electrophoresis, the analysis conditions were optimized at different pH values and at different background electrolyte (BGE) concentrations. The best separation results were obtained using a buffer containing 25 mM sodium monophosphate, the pH of which was adjusted to 2.5 with 0.1 M phosphoric acid.

**Figure 2:**
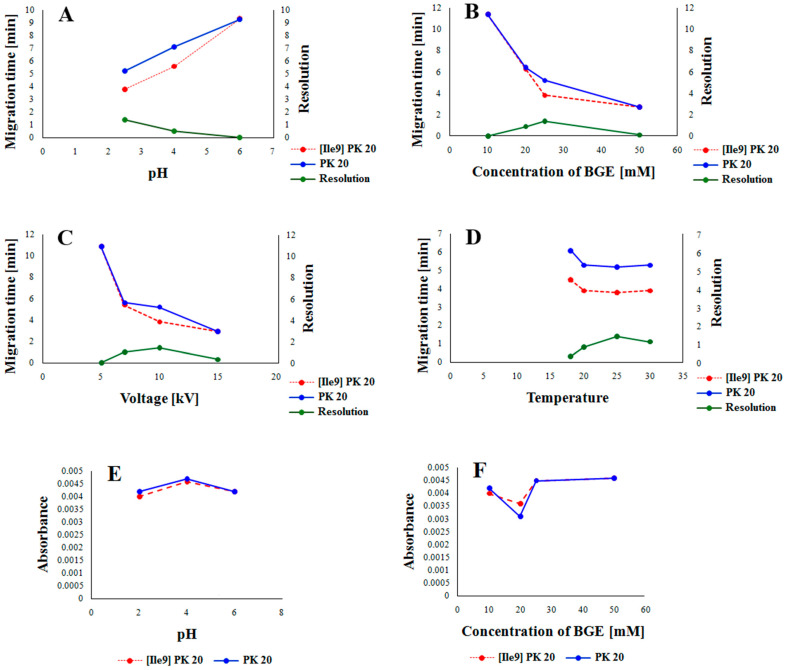
The influence of pH (**A**), BGE concentration (**B**), applied voltage (**C**), and capillary temperature (**D**) on the resolution of [Ile9]PK20 and PK20 and the influence of pH (**E**) and concentration of the buffer (**F**) on the absorbance value of [Ile9]PK20 and PK20.

**Figure 3:**
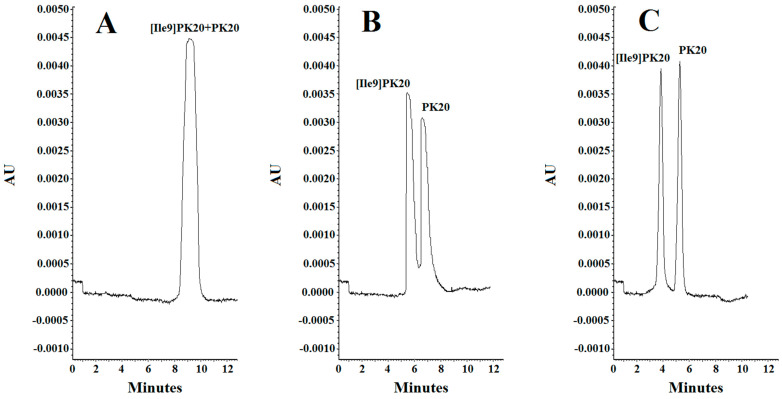
Separation of PK20 and [Ile9]PK20 at a concentration of 500 ng/mL under different analysis conditions: (**A**) 10 mM phosphate buffer, pH = 6.0, voltage 5 kV, temperature 18 °C; (**B**) 20 mM phosphate buffer, pH = 4.0, voltage 7 kV, temperature 20 °C; (**C**) 25 mM phosphate buffer, pH = 2.5, voltage 10 kV, temperature 25 °C.

In order to develop the best conditions for studying the compounds [Ile9]Pk20 and PK20 using the CE technique, preliminary studies were carried out under the influence of factors such as pH, buffer concentration, voltage, and temperature to assess the separation capabilities of both compounds. Indeed, for example, temperature is a well-known factor affecting both efficiency and selectivity in CE [11,12]. This, in turn, was found to be dependent on the type of buffer used, as low-pH buffer ions have positive temperature dependence; high-pH buffer ions have a negative one [11]. Also, the abrupt application of high electric field strength may lead, in some cases, to lower separation efficiency [13]. In this aspect, the influence of all the abovementioned parameters on the resolution is presented in Figure 2. The graphical representation of the influence of pH and buffer concentration on the absorbance value of both compounds is presented in Figure 3.

In this study, we examined the influence of parametric factors (Figure 2) on the optimal separation conditions for the peptides under study. As shown in Figure 2, all parameters tested (pH, buffer concentration, voltage, and temperature) influenced the separation process. Based on the analysis, it appears that the most important factor affecting the migration rate and achieving the best resolution was buffer pH. Lowering this pH contributed to increased protonation of the peptides, thus accelerating their migration and improving resolution (Figure 2A). Buffer concentration also influenced the migration rate and resolution. Low buffer concentrations reduced the migration rate and resolution of the peptides under study. Only a 25 mM concentration allowed for achieving adequate resolution and optimally short migration times. After exceeding 25 mM, the migration rate increased at the expense of reduced resolution (Figure 2B). A similar situation occurred when examining the effect of voltage on the migration rate and resolution. Here, too, a range of low voltage values resulted in the slow migration of molecules without providing adequate resolution. However, increasing the voltage to 10 kV reduced the migration time and improved the resolution. Further increases in voltage significantly worsened the resolution (Figure 2C). The temperature only initially had a significant effect on the peptide migration rate. Only the initial increase in temperature accelerated the migration of molecules and improved the resolution (Figure 2D). The influence of pH and buffer concentration was also evident when examining their effect on absorbance values. Both low (pH = 2.5) and high (pH = 6.5) pH values slightly decreased the absorbance values of the tested components, but only at low pH values was adequate resolution of the tested peptides achieved (Figure 2E). A very similar situation occurred when determining the effect of buffer concentration on absorbance values. As can be seen, both low and high concentrations contributed to increased absorbance values, but only at a concentration of 25 mM was adequate resolution of the tested components achieved (Figure 2E).

The effect of concentration of the background electrolyte (BGE) was analyzed in a range from 10 mM to 25 mM. At a concentration of 10 mM, the obtained peak shapes were unsatisfactory, and the separation of the studied compounds PK20 and [Ile9]PK20 was not achieved at this concentration (Figure 3A). This can result from the fact that the effective mobilities of the compounds tested approach each other. Notably, an appropriate resolution of the components is crucial for achieving reliable results. Therefore, by changing the concentration, the optimal separation of compounds was sought. At a buffer concentration of 20 mM, a clear partial separation of the compounds PK20 and [Ile9]PK20 was obtained (Figure 3B). However, the best results in terms of separation efficiency and analysis time were obtained using a buffer concentration of 25 mM with pH 2.5 (Figure 3C).

This nicely shows the salt effects of the buffer upon the degrees of ionization of the two hybrid compounds that lead to a convergence of their electrophoretic mobilities.

Resolution factors were also calculated for neighboring peaks. In the case of parameters presented in Figure 3B, due to insufficient separation, it was not possible to determine the resolution value. However, for the conditions shown in Figure 3C, the resolution (Rs) was 1.4, which clearly indicates a clear improvement in separation. The resolution calculations were calculated according to the following formula: Rs = 2(tm_2_ − tm_1_)/(Wb_1_ + Wb_2_). The best results were obtained at a buffer concentration of 25 mM (Figure 3C).

Considering parameters such as resolution, peak shape, and migration times of PK20 compounds and its analogue [Ile9]PK20, the final conditions for the background electrolyte (BGE) were set to a 25 mM concentration and pH 2.5. The average detection times for both compounds were 3.80 and 5.20 min, respectively (Figure 3C). Figure 3 compares the electrophoretic separation of PK20 and [Ile9]PK20 under different conditions, including different buffer pH values, concentration, applied voltage, and temperature. A moderate buffer concentration (25 mM phosphate) provided a balance between current stability and efficient separation, minimizing Joule heating. Increasing the separation voltage from 5 to 10 kV significantly shortened the migration time without compromising the resolution. Furthermore, increasing the capillary temperature from 15 °C to 25 °C reduced the buffer viscosity, allowing for faster analyte migration and further reducing the analysis time. The improved peak shape or reduced migration time shown in Figure 3C compared with Figure 3A,B was likely due to a temperature-induced reduction in the viscosity of the separation buffer; this is commonly reported by others [14]. Together, these optimized parameters contributed to sharper peak shapes, improved resolution (Rs = 1.4), and reduced overall analysis time, which demonstrates the robustness and efficiency of the developed method.

**Figure 4:**
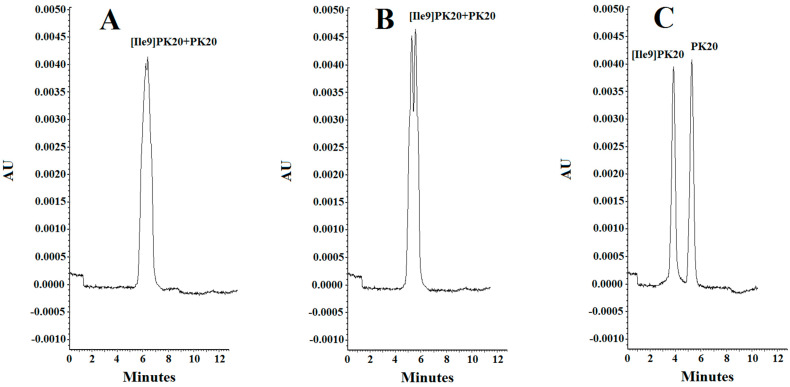
Separation of PK20 and [Ile9]PK20 at a concentration of 500 ng/mL under different pH conditions: (**A**) 25 mM phosphate buffer, pH = 6.0, voltage 5 kV, temperature 18 °C; (**B**) 25 mM phosphate buffer, pH = 4.0, voltage 7 kV, temperature 20 °C; (**C**) 25 mM phosphate buffer, pH = 2.5, voltage 10 kV, temperature 25 °C.

The effect of the pH of the background electrolyte (BGE) was analyzed in a range from 6.0 to 2.5. At pH 6.0, the obtained peak shapes were unsatisfactory, and the separation of the studied compounds PK20 and [Ile9]PK20 was not achieved at this pH value (Figure 4A). This can result from the fact that the effective mobilities of the compounds tested approach each other. Notably, an appropriate resolution of the components is crucial for achieving reliable results. Therefore, by changing the pH, the optimal separation of compounds was sought. It was observed that the resolution of the studied compounds increased with decreasing buffer pH, at which it reached a maximum, which is consistent with the literature data [15]. At buffer pH 4.0, a clear, although only partial, separation of the compounds PK20 and [Ile9]PK20 was obtained (Figure 4B). However, the best results in terms of separation efficiency and analysis time were obtained using a buffer with pH 2.5 (Figure 4C).

The effect of applied voltage on separation efficiency and migration time was evaluated at 5, 7, and 10 kV. Increasing the voltage to 10 kV resulted in improved resolution, as shown in Figure 2. However, after exceeding 10 kV, a decrease in resolution was observed, although the migration time continued to shorten with increasing voltage. Indeed, it is well established that an excessively increased voltage adversely affects the resolution [16]. In line with this, the voltage range of 5–10 kV is usually used in CE techniques [17,18], which nicely corresponds to our study.

**Figure 5:**
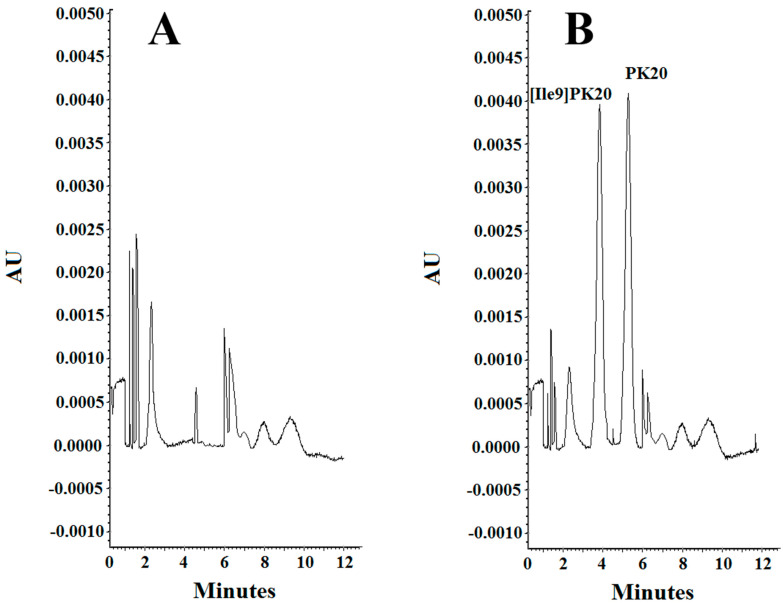
Specificity of the method. (**A**) Blank serum and (**B**) serum sample containing PK20 and [Ile9]PK20 at a concentration of 500 ng/mL under the following separation conditions: 25 mM phosphate buffer, pH = 2.5, voltage 10kV, temperature 25 °C.

In the case of temperature influence, three different values were used, such as 18, 20, and 25 °C, respectively (Figure 2). It was shown that increasing the temperature contributed to the improvement of resolution. The best results were obtained at 25 °C. This seems to be quite unexpected, as the increase in temperature is generally responsible for decreasing resolution in CE [19]. Obviously, several important reasons can exist, among which the temperature-induced denaturation of proteins/peptides is of significant importance [20,21]. However, for both peptides investigated, it was recently revealed that they are quite stable under thermal conditions (i.e., from −80 to +37 °C) [22]. In summary, since the best separation of [Ile9]PK20 and PK20 compounds was obtained using a 25 mM phosphate buffer at pH 2.5, as well as a separation voltage of 10 kV and a temperature of 25 °C, the developed method was used for preliminary studies of these compounds and their derivatives in serum (Figure 5).

### 2.2. Method Development

Capillary electrophoresis (CE) enables the selective monitoring of PK20 and [Ile9]PK20 compounds and eliminates interference from endogenous components that may co-elute from serum samples. The separation of PK20 and its modified analogue [Ile9]PK20 was performed using 25 mM phosphate buffer at pH 2.5. In order to develop the method and determine the validation parameters, the following elements were optimized: buffer type (buffer H), BGE concentration, composition and concentration of the extraction phase, wavelength, voltage, and temperature. The extraction procedure was performed according to the method described by Chertov et al. [23] but in a simplified form, as presented below.

In this study, phosphate buffer containing 25 mM sodium monophosphate was selected as the background electrolyte (BGE) due to its high separation efficiency, which was previously demonstrated. Considering the analysis time and separation quality, +10 kV was selected as the separation voltage. Based on the conducted studies, the separation was performed by capillary electrophoresis (CE) under the following conditions: BGE—25 mM phosphate buffer, pH 2.5; voltage +10 kV; detection temperature 25 °C; and wavelength 200 nm. Measurements were performed using a fused silica capillary with an effective length of 20 cm and a diameter of 75 µm. Samples were introduced into the capillary by hydrodynamic injection. Under the described conditions, PK20 and [Ile9]PK20 compounds were separated and migrated in less than 6 min. Peak identification was performed by separately injecting standards and comparing migration times.

### 2.3. Method Validation

Linearity, precision, specificity, accuracy, carry-over, extraction recoveries, and the limit of detection (LOD) and quantification (LOQ) of the developed method were validated according to the guidelines of the International Council for Harmonization (ICH) [24]. The first step preceding the validation was to search for optimal buffer concentrations, as described in Section 3.1.

**Figure 6:**
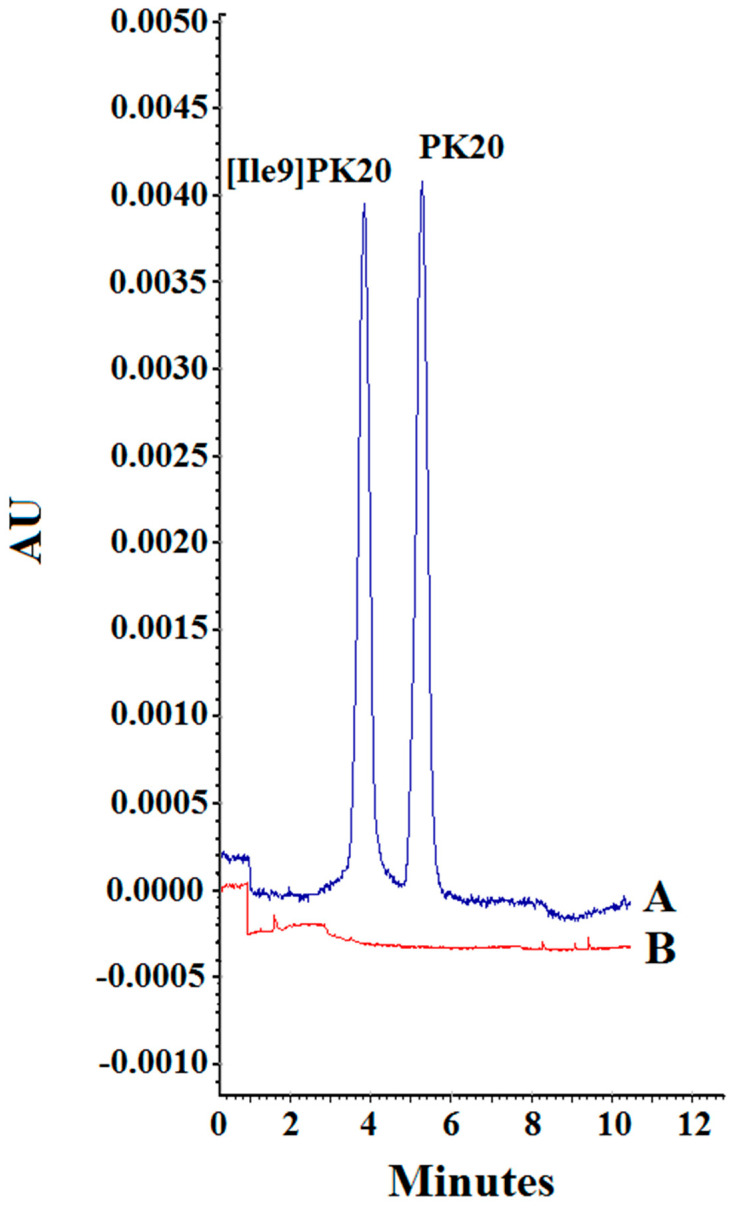
The carry-over effect. (A) PK20 and [Ile9]PK20 at a concentration of 500 ng/mL. (B) Blank sample.

The specificity of the method was defined as the ability to distinguish and determine the analyte from the endogenous components of the matrix or other contaminants present in the sample. The presence of possible endogenous interferences was assessed by analyzing 10 serum samples spiked with PK20 and [Ile9]PK20 compounds. The measurement results (Figure 6) showed no endogenous interference for each of the tested analytes.

The limits of detection (LODs) and limits of quantification (LOQs) for the chimeric compounds PK20 and [Ile9]PK20 were determined based on a series of analyses performed on human serum samples spiked with decreasing concentrations of each analyte. These parameters were calculated in accordance with the guidelines set forth by the International Council for Harmonisation (ICH), ensuring methodological consistency and regulatory compliance.

Specifically, the LOD was defined as the lowest concentration at which a signal-to-noise ratio (S/N) of at least 3:1 was observed, indicating reliable analyte detection above baseline noise. In turn, the LOQ corresponded to the minimum concentration at which the analyte could be quantitatively measured with acceptable levels of precision and accuracy, defined as a coefficient of variation (CV%) not exceeding 20%. All quantitative analyses were conducted using 100 µL aliquots of human serum spiked with known amounts of each compound.

The method demonstrated excellent linearity across the tested concentration range, as evidenced by the coefficients of determination (R^2^) for the calibration curves, which ranged from 0.9991 to 0.9998. Based on these data, the LODs for PK20 and [Ile^9^]PK20 were estimated at 10.75 ng/mL and 10.83 ng/mL, respectively, while the LOQs were established at 34.72 ng/mL and 34.98 ng/mL (Table 1), confirming the high sensitivity of the developed analytical procedure.

To assess method recovery, serum samples were spiked with the target analytes at three concentration levels—low, medium, and high—and subsequently subjected to the complete extraction and analytical workflow. The recovery was calculated by comparing the mean relative peak areas of the extracted samples with those of pure standard solutions and with samples spiked post-extraction. This approach provided insight into both the extraction efficiency and matrix effects. The recovery values obtained for both compounds were satisfactory, ranging from 94.8% to 100.0%, indicating that the sample preparation protocol did not result in significant analyte loss (Table 2). The precision of the proposed method in terms of repeatability was determined by conducting a replicate analysis (n = 6) of serum extracts that were spiked with 20 ng/mL, 500 ng/mL, and 5000 ng/mL of each compound.

Similarly, the relative standard deviation (RSD) for migration time and relative peak area was less than 3.0% (Table 3). Stability tests were performed under high-quality-control conditions.

The repeatability (intra-day precision) of the method was evaluated by analyzing six replicate serum samples spiked with each compound at three representative concentration levels, namely 20 ng/mL, 500 ng/mL, and 5000 ng/mL. The method exhibited high precision, with relative standard deviations (RSDs) for both migration times and relative peak areas remaining below 3.0% across all concentrations (Table 3). These results confirm the robustness and reproducibility of the analytical procedure under routine laboratory conditions.

To ensure the applicability of the method for bioanalytical studies, stability tests were conducted under various storage and handling conditions. The analytes were found to be stable in serum at room temperature for at least 6 h, and no significant degradation was observed. Additionally, short-term stability was confirmed by storing serum samples at −25 °C for 30 days. Analytical performance after storage showed a significant, measurable loss of compound integrity of 52.16–75.90% (Table 4).

Finally, potential carry-over effects (Figure 6) were evaluated to ensure that no analyte residue persisted in the capillary or detection system following high-concentration injections. Blank serum samples were analyzed immediately after the injection of samples containing 500 ng/mL of each analyte. No detectable peaks were observed in the blanks, confirming the absence of carry-over and further supporting the reliability of the method for sequential analyses.

These results are somehow in good agreement with our previous study, indicating significant stability of the chimeras in biological liquids of human blood, in particular the PK20 opioid–neurotensin hybrid peptide. In fact, this compound was found to be enzymatically stable in human plasma; the exact half-life of the peptide was calculated to be 31 h 45 min [25].

## 3. Materials and Methods

### 3.1. Chemicals and Reagents

The synthesis of both opioid–neurotensin compounds was performed as previously described [8] using the solid-phase peptide synthesis method. Sodium dihydrogen phosphate, phosphoric acid, acetonitrile, and trifluoroacetic acid were purchased from Sigma Aldrich (Darmstadt, Germany). The serum standard Bovprec Control 2 was purchased from Randox (Warsaw, Poland).

### 3.2. Instrumentation

A Beckman Coulter P/ACE MDQ CE system equipped with an autosampler and UV/VIS detector was used. Data were acquired using Karat software v.32. The eCAP fused silica capillary (Beckman Coulter, Brea, CA, USA) had a total length of 30 cm, effective length of 20 cm, i.d. of 75 µm, and o.d. of 375 µm.

### 3.3. Sample Preparation

The stock solutions of PK20 and [Ile9]PK20 compounds at a concentration of 10,000 ng/mL were prepared by dissolving the appropriate amounts of the substances in a 25 mM phosphate buffer at pH 2.5. Calibration solutions at specific concentrations were prepared on their basis. All solutions were stored at −15 °C. Samples for the preparation of working standards and quality control were prepared on the basis of standard serum to which the appropriate amounts of PK20 and [Ile9]PK20 compounds had been previously added. Calibration solutions at concentrations of 10, 50, 100, 500, 1000, and 5000 ng/mL were prepared in both water and serum by adding appropriate volumes of the stock solutions to serum samples free of the tested analytes. Standard serum served as the dilution medium and did not contain PK20 or [Ile9]PK20 compounds.

To a 100 µL serum sample, 200 µL of acetonitrile (ACN) containing 0.1% trifluoroacetic acid (TFA) was added then immediately mixed by vortexing and centrifuged at 20,000× *g* for 4 min. The resulting supernatant was filtered using an ultrafiltration centrifuge (MWCO 30,000; Microcon YM-30, Millipore Corporation, Bradford, MA, USA) and then lyophilized. The dry residue was dissolved in 100 µL of deionized water and introduced into the capillary. The extraction procedure for PK20 and [Ile9]PK20 compounds was performed for three concentration ranges, namely 20 ng/mL, 500 ng/mL, and 5000 ng/mL.

### 3.4. CE Conditions

Electrophoretic separations were performed in an eCAP capillary made of fused silica with a total length of 30 cm, an effective length of 20 cm, an internal diameter of 75 µm, and an external diameter of 375 µm. For the analysis of PK20 and [Ile9]PK20 compounds, sodium monophosphate (25 mM) was used as a background electrolyte (BGE), the pH of which was adjusted to 2.5 using phosphoric acid. The detector parameters were set as follows: wavelength—200 nm; temperature—25 °C; and voltage—+5 kV. New capillaries were conditioned by rinsing them successively with 1 M NaOH (15 min, 10 psi), water (15 min, 10 psi), and BGE (20 min, 10 psi). Then, the system was electroconditioned with a working buffer, applying a separation voltage of +5 kV for 20 min. Between each day of work, the capillary was conditioned by rinsing successively with 0.1 M NaOH (5 min, 10 psi), water (5 min, 10 psi), and BGE (10 min, 10 psi).

Samples were introduced into the system hydrodynamically at a pressure of 2 psi for 6 s. Electrophoresis was performed at a voltage of 10 kV (normal polarity, anode on the inlet side of the capillary) at a temperature of 25 °C. Between subsequent analyses, the capillary was rinsed with water for 2 min and with BGE for 3 min. After each series of analyses, the working buffer was replaced to ensure repeatable separations.

After extraction and dissolution of the samples in 0.1 mL of aqueous solution, the samples were automatically injected under pressure (3 psi for 10 s). In the evening, the vials containing the anode and cathode buffer were emptied, and before the start of the next day of work, the working buffer was refilled.

## 4. Conclusions

The developed capillary electrophoresis method enables a simultaneous determination of hybrid opioid–neurotensin peptides PK20 and [Ile^9^]PK20 in both aqueous solutions and biological material such as blood serum. This method is characterized by high resolution, sensitivity, and repeatability, which makes it suitable for bioanalytical applications, including pharmacokinetic and bioavailability studies.

The high efficiency of compound separation in a short analysis time while using small sample volumes confirms the effectiveness and economy of the method. The advantage of the developed approach is also demonstrated by the good linearity of calibration curves, the low value of the limits of quantification (LOD) and quantification (LOQ), and the lack of carry-over effect.

All these features confirm the usefulness of the method as a precise and reliable tool for the quantitative determination of chimeric therapeutic peptides in preclinical studies.

## Figures and Tables

**Table 1:** Regression equation and limits of detections and quantification for compounds PK20 and [Ile9]PK20.

Compound	Linearity Range	R^2^	(RSD) [%]	LOD	LOQ	Regression Equation	(±SD)	
	ng/mL			ng/mL	ng/mL		Slope	Intercept
PK20	50–5000	0.9991	2.26	10.75	34.72	y = 34.464x − 319.69	±0.23	±1.02
[Ile9]PK20	50–5000	0.9999	1.98	10.83	34.98	y = 32.453x − 54.352	±0.19	±0.78

**Table 2:** Recovery data of compounds PK20 and [Ile9]PK20 (n = 6).

Compound	Added Amount (ng/mL)	Observed Amount Mean ± SD (ng/mL)	% Recovery	%RSD
PK20	50	48.96 ± 1.3	94.8	6.85
	500	497.50 ± 1.9	99.5	0.38
	5000	5000.00 ± 0.7	100.0	0.014
[Ile9]PK20	50	49.22 ± 1.1	96.1	5.72
	500	499.00 ± 1.3	99.8	0.26
	5000	5000.00 ± 0.5	100.0	0.010

**Table 3:** Intra-day and inter-day precision of the tested PK20 and [Ile9]PK20 hybrid peptides.

Compound	Concentration (ng/mL)	Intra-Day Precision (n = 6, mean)					
		Day 1		Day 2		Day 3	
		m_t_/%RSD	p_a_/%RSD	m_t_/%RSD	p_a_/%RSD	m_t_/%RSD	p_a_/%RSD
PK20	50	3.72/0.9	325/2.3	3.78/0.8	305/2.7	3.75/0.8	317/2.4
	500	3.68/0.5	8240/1.2	3.70/0.6	8190/1.4	3.70/0.5	8200/1.3
[Ile9]PK20	50	4.25/0.8	361/2.1	4.21/0.9	345/2.4	4.24/0.8	358/2.1
	500	4.20/0.5	9100/1.0	4.18/0.7	9003/1.6	4.20/0.6	9087/1.2
**Compound**	**Concentration** **ng/mL**	**Inter-Day Precision (n = 18, mean)**					
		**m_t_/%RSD**			**p_a_/%RSD**		
PK20	50	3.75/0.6			320/2.3		
	500	3.72/0.6			8250/1.3		
[Ile9]PK20	50	4.19/0.5			360/2.1		
	500	4.20/0.3			9140/1.1		

m_t_—migration time; p_a_—peak area.

**Table 4:** Stability studies for compounds PK20 and [Ile9]PK20.

Compound	Spiked Concentration (ng/mL)	BenchTop ^a^ Obtained Concentration (ng/mL) ^d^		Freeze and Thaw ^b^ Obtained Concentration (ng/mL) ^d^	
		Mean ± SD	%	Mean ± SD	%
PK20	50	23.92 ± 1.2	47.84	12.05 ± 1.5	24.10
	500	240.53 ± 0.9	48.11	126.20 ± 0.7	25.24
[Ile9]PK20	50	24.46 ± 1.1	48.92	12.72 ± 1.4	25.44
	500	246.15 ± 1.0	49.23	127.53 ± 1.2	25.51
**Compound**	**Spiked** **Concentration** **(ng/mL)**	**Short Term ^c^**			
		**Obtained Concentration (ng/mL) ^d^** **Mean ± SD**		**%**	
PK20	50	47.52 ± 1.4		95.04	
	500	495.58 ± 1.1		99.12	
[Ile9]PK20	50	48.85 ± 1.2		97.70	
	500	497.80 ± 1.1		99.56	

^a^ After 6 h at room temperature. ^b^ After three freeze and thaw cycles at −20 °C ^c^. At −25 °C for 30 days. ^d^ Values are mean ± SD for five replicates.

## Data Availability

The original contributions presented in this study are included in the article. Further inquiries can be directed to the corresponding author.
